# SARS-CoV-2 Viral Load in the Pulmonary Compartment of Critically Ill COVID-19 Patients Correlates with Viral Serum Load and Fatal Outcomes

**DOI:** 10.3390/v14061292

**Published:** 2022-06-14

**Authors:** Mario Ynga-Durand, Henrike Maaß, Marko Milošević, Fran Krstanović, Marina Pribanić Matešić, Stipan Jonjić, Alen Protić, Ilija Brizić, Alan Šustić, Luka Čičin-Šain

**Affiliations:** 1 Helmholtz Center for Infection Research, Department of Viral Immunology, 38124 Braunschweig, Germany; marioalberto.yngadurand@helmholtz-hzi.de (M.Y.-D.); henrike.maass@helmholtz-hzi.de (H.M.); 2 Department of Anesthesiology, Reanimation, Intensive Care and Emergency Medicine, Faculty of Medicine, University of Rijeka, 51000 Rijeka, Croatia; miloshevic.marko@gmail.com (M.M.); alen.protic@medri.uniri.hr (A.P.); asustic@medri.uniri.hr (A.Š.); 3 Center for Proteomics, Faculty of Medicine, University of Rijeka, 51000 Rijeka, Croatia; fran.krstanovic@medri.uniri.hr (F.K.); marina.pribanic.matesic@medri.uniri.hr (M.P.M.); stipan.jonjic@medri.uniri.hr (S.J.); ilija.brizic@medri.uniri.hr (I.B.); 4 Department of Clinical Medical Science II, Faculty of Health Studies, University of Rijeka, 51000 Rijeka, Croatia; 5 German Centre for Infection Research (DZIF), Partner Site Hannover/Braunschweig, 38124 Braunschweig, Germany; 6 Centre for Individualised Infection Medicine (CiiM), A Joint Venture of Helmholtz Centre for Infection Research and Hannover Medical School, 30625 Hannover, Germany

**Keywords:** SARS-CoV-2, COVID-19, bronchoalveolar lavage, qPCR, critical care

## Abstract

While SARS-CoV-2 detection in sputum and swabs from the upper respiratory tract has been used as a diagnostic tool, virus quantification showed poor correlation to disease outcome and thus, poor prognostic value. Although the pulmonary compartment represents a relevant site for viral load analysis, limited data exploring the lower respiratory tract is available, and its association to clinical outcomes is relatively unknown. Using bronchoalveolar lavage (BAL) and serum samples, we quantified SARS-CoV-2 copy numbers in the pulmonary and systemic compartments of critically ill patients admitted to the intensive care unit of a COVID-19 referral hospital in Croatia during the second and third pandemic waves. Clinical data, including 30-day survival after ICU admission, were included. We found that elevated SARS-CoV-2 copy numbers in both BAL and serum samples were associated with fatal outcomes. Remarkably, the highest and earliest viral loads after initiation of mechanical ventilation support were increased in the non-survival group. Our results imply that viral loads in the lungs contribute to COVID-19 disease severity, while blood titers correlate with lung virus titers, albeit at a lower level. Moreover, they suggest that BAL SARS-CoV-2 copy number quantification at ICU admission may provide a predictive parameter of clinical COVID-19 outcomes.

## 1. Introduction

Severe acute respiratory syndrome coronavirus-2 (SARS-CoV-2), the causative agent of COVID-19 [1], was detected for the first time in Wuhan, China at the end of 2019 [2]. The virus spread rapidly around the globe and the World Health Organization (WHO) declared a pandemic on 11 March 2020 [3]. From early on, it was reported that the COVID-19 clinical spectrum varies broadly from asymptomatic to acute respiratory failure leading to death [4]. Variables such as comorbidities and innate immunity defects have been identified as risk factors for severity and mortality [5,6,7], especially in older populations. European countries with elderly demographics were severely hit [8], especially during periods of exponential increase, also denominated pandemic “waves”. In Croatia, a second and a third wave were identified from October 2020 to December 2020, and from February 2021 to May 2021, respectively [9,10]. These waves were associated with increased mortality and healthcare burden, with a peak in intensive care unit (ICU) admissions associated to COVID-19.

While previous studies have reported analysis of SARS-CoV-2 RNA load in various body fluids from hospitalized COVID-19 patients [11,12,13], its use as a tool for severity assessment or prognosis remains limited. Multiple reports have described the association of the serum SARS-CoV-2 viral copy number with outcomes, but scarce information on the lung compartment is available. Importantly, it is proposed that initial alveolar SARS-CoV-2 replication precedes the alveolar–vascular barrier breakdown which permits the systemic access of viral RNA and proteins that may contribute to disease severity and complications [14]. Therefore, SARS-CoV-2 viral load in the pulmonary compartment may represent a valuable site for investigating COVID-19 severity association and/or outcome prediction. To determine the viral RNA load association to COVID-19 clinical outcomes in a high mortality cohort, we decided to prospectively follow a COVID-19 critical care patient cohort from the Clinical Hospital Center Rijeka in Croatia during the second and third pandemic waves, determining viral RNA dynamics on both the systemic and pulmonary compartments. We show here that the virus can be quantified in the bronchoalveolar lavage (BAL) and serum in the vast majority of severely ill COVID-19 patients, that the two values correlated to each other, and that they were elevated in the patients that passed away. 

## 2. Materials and Methods

### 2.1. Study Participants

The study population included 54 patients admitted to the COVID-19 Intensive Care Unit (ICU) of the Clinical Hospital Center Rijeka with a diagnosis of severe COVID-19 [15] and acute respiratory distress syndrome defined by the Berlin criteria [16]. These patients required invasive ventilatory support, and ICU specialists followed standardized therapeutic guidelines. Thirty-three patients were sampled from November until December of 2020, and 21 patients from March until April 2021. A non-critically ill group was included, composed of 18 patients with severe COVID-19 (hospitalized oxygen-dependent SARS-CoV-2 positive patients without invasive respiratory or hemodynamic support requirements) and 4 non-hospitalized SARS-CoV-2 positive patients (identified by screening). All patients included in this study were initially diagnosed with SARS-CoV-2 infection by RT-qPCR testing from nasopharyngeal swabs. A highly standardized COVID-19 management protocol was followed in all ICU patients described in Supplementary File S1.

### 2.2. Clinical Data and Outcomes

Clinical data were recollected from the electronic medical files from the Clinical Hospital Center Rijeka, omitting identifier information to protect patient privacy. Clinical characteristics of ICU survivors and non-survivors are listed in Table 1. Two of 18 patients from the non-ICU symptomatic group did not have complete/available clinical records and were not included in Table 1. Disease severity scoring at admission was evaluated by an intensive care specialist.

### 2.3. Samples Collection

#### 2.3.1. Bronchoalveolar Lavage (BAL) Samples

BAL samples from ICU patients were obtained as a routine procedure for microbiological analysis as follows: within the first 36 h after intubation, 10 mL of sterile saline was instilled in the main right bronchus through the endotracheal tube (ET). Fluid was collected by aspiration, introducing an aspiration cannula 10-15 cm through the ET until at least 5 mL of aspirate was obtained. Samples were collected every three days until the 10th day of ICU stay (except ICU discharge or death) and transported to the Center for Proteomics of the University of Rijeka for further processing. Specimens were filtered through a 100 µm cell strainer to remove mucous strands and centrifuged at 400× *g* at 4 °C for 7 min. The supernatant was aliquoted and stored at −80 °C. The samples were shipped on dry ice to the Helmholtz Center for Infection Research in Braunschweig and stored at −80 °C. 

#### 2.3.2. Serum Samples

Blood was drawn from all patients on the same day as the BAL procedure was performed and processed at the Center for Proteomics at the University of Rijeka. Whole blood was incubated at room temperature for 30–60 min until a blood clot was visible. Serum and blood clot were separated and the serum was centrifuged at 1500× *g* for 10 min. Serum was aliquoted and stored at −20 °C. The samples were shipped on dry ice to the Helmholtz Center for Infection Research in Braunschweig and stored at −80 °C.

### 2.4. Isolation and Quantification of Viral RNA from BAL and Serum Samples

BAL samples were filtered through a 70 µm filter and centrifuged at 800× *g* and 4 °C for 7 min before isolating RNA. Serum samples were preprocessed by centrifugation at 800× *g* and 4 °C for 7 min. Isolation of RNA was performed with 200 µL of the according supernatant using the Innuprep Virus DNA/RNA virus kit (Analytic Jena, Jena, Germany, Cat # 845-KS-4710250) according to manufacturer’s instructions. Viral RNA was transcribed into cDNA followed by a PCR amplification. Primers and probes target the genome region coding for the nucleocapsid protein N2 of SARS-CoV-2 or the host housekeeping gene RNase P. A 1:10 serial dilution of a control plasmid (Integrated DNA Technologies, Coralville, IA, USA, Cat # 10006625) was measured simultaneously for quantification of the isolated RNA. Each reaction mixture contained 8.5 µL nuclease-free water, 1.5 µL combined Primer/Probe Mix (Integrated DNA Technologies Cat # 10006713), 5 µL TaqPathTM 1-Step RT-qPCR Master Mix (4×) (Thermo Fisher, Waltam, MA, USA, Cat A15300) and 5 µL isolated RNA or control plasmid. The qPCR was run in a Real Time PCR 7500 FAST system in triplicates (Thermo Fisher Scientific, Waltam, MA, USA). The following cycling conditions were used: one cycle at 25 °C for 2 min, one cycle at 50 °C for 15 min, one cycle at 95 °C for 2 min and 40 cycles of 95 °C for 3 s and 60 °C for 30 s. Viral Copy number calculation was performed by OneStep qPCR Software (Thermo Fisher Scientific, Waltam, MA, USA). For analysis purposes, the first sample of each patient was defined as the earliest sample. The sample with the highest viral load of all samples per patient was defined as highest sample. 

#### Identification of SARS-CoV-2 Variants

SARS-CoV-2 variants were identified with the GSD NovaType II SARS-CoV-2 RT-PCR assay (PCOV6083T, Gold Standard Diagnostics Europe, Dietzenbach, Germany). This assay allows for the simultaneous detection of the SARS-CoV-2 mutations K417N, E484K and N501Y within the spike gene, and was conducted following the manufacturer’s instructions. Detection of the amplified viral nucleic acid fragments was performed using the LightCycler^®^ 480 (Roche, Basel, Switzerland) using the extracted RNA with the highest quantification of viral copy number per patient, in technical duplicates. As a positive control, a control plasmid containing the SARS-CoV-2 S gene variants sequence, as well as RNA extracted from sequenced SARS-CoV-2 variant isolates (alpha and beta) were used. Two patients’ variant identifications were considered inconclusive after repeated measurements of viral RNA extracted from different timepoints. These two patients were survivors and had lower viral loads in comparison to the rest of the cohort (one of them has the lowest viral copy number quantification of the cohort). This may explain the difficulties in variant detection using this assay. 

### 2.5. Statistical Analysis

A logarithmic normalization was performed on viral RNA copy number per mL. Fisher’s exact test was applied for comparison between categorical variables. Shapiro–Wilks test was used as a normality test. Depending on the results, parametric or non-parametric analysis was used as follows: one-way ANOVA with Bonferroni correction for multiple comparisons, unpaired T test with Welch’s correction or the Mann–Whitney test was used for comparisons where appropriate. Third order polynomial non-linear regression was performed for trend representation. For multivariate analysis, clinical variables that differed between survivors and non-survivors at admission were included, as well as viral copy numbers. ROC analysis and Youden’s J statistics were used to identify optimal cut-off thresholds using MedCalc Statistical Software version 19.2.6 (MedCalc Software, Ostend, Belgium). Then, multiple logistic regression was performed after categorical conversion of the dataset. GraphPad Prism version 9.02 (GraphPad Software, La Jolla CA, USA) was used for statistical analysis and graphing. 

### 2.6. Ethical Considerations

The study protocol was approved by the Institutional Review Board of the Rijeka Clinical Hospital Center (2170-29-02/1-20-2). Written informed consent by the patient or surrogate was waived by the Ethics Committee of the Rijeka Clinical Hospital Center, as the sampling was a part of standard diagnostic monitoring.

## 3. Results

### 3.1. Demographic and Clinical Characteristics of the Study Population

Fifty-four patients were included; their main demographics and baseline clinical characteristics are shown in Table 1, including laboratory data and comorbidities. No significant difference in comorbidities was found between the survival and non-survival groups. The non-survival group was older and scored higher on ICU severity score at admission (SOFA and APACHE II). The survival group had a longer ICU stay and at admission presented lower C Reactive Protein (CRP) levels. During ICU stay, the non-survival group presented a higher percentage of shock and pharmacological hemodynamic support. No significant differences in days of mechanical ventilation or ventilator-associated pneumonia (VAP) were found. 

Relevantly, as part of the standardized management guidelines, the entire ICU group (survival and non-survival) received a systemic steroid, while none received convalescent plasma therapy. Regarding time since onset of symptom to first sample taken, only reliable and consistent information from the patient, caretaker or surrogate was included (40 out of 54 patients). No significant differences were found between the survival and non-survival group (median 12.5 vs. 10, respectively). 

We received samples from patients admitted during the second and third pandemic waves. Relevant clinical characteristics are shown in Table 2. The groups were similar in their demographic characteristics and baseline respiratory compromise. Organ failure scoring (SOFA) was slightly higher in the third wave group (median difference = 1). 

### 3.2. SARS-CoV-2 Viral Load in Pulmonary and Systemic Compartments

**Figure 1:**
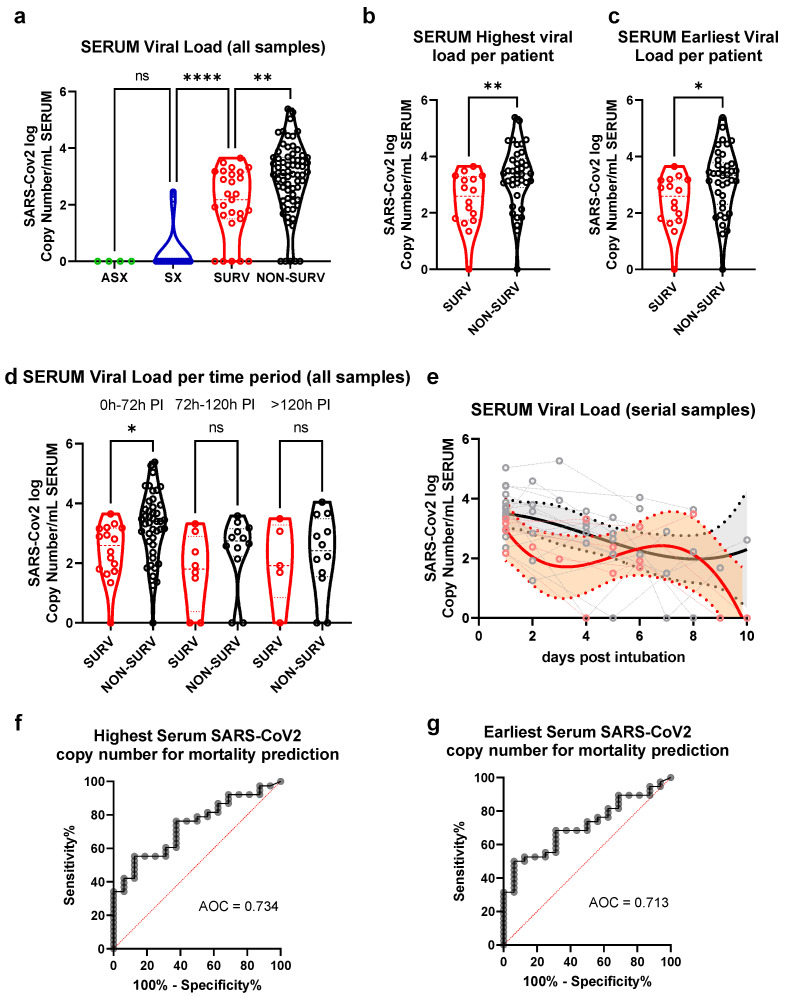
SARS-CoV-2 viral load in serum samples from SARS-CoV-2 patients. Serum was obtained from patients admitted to the ICU with COVID-19 associated acute respiratory failure diagnosis divided by their mortality outcome (SURV survival, NON-SURV non-survival), a hospitalized non-ICU group on supplemental oxygen “SX”, and a non-hospitalized group “NH”. In some ICU patients, serum sampling was performed at multiple time points. (**a**) Violin plots of median and quartile SARS-CoV-2 copy numbers of individual samples pooled from all time points post-intubation are shown. Circles show data for each patient. Asterisks indicate significant differences between groups according to one-way ANOVA followed by Bonferroni post-analysis. (**b**) Student t-test analysis of ICU samples with the highest viral load per patient and (**c**) earliest sample available per patient. (**d**) A pooled analysis divided by sampling period after intubation (PI) was performed by ANOVA and Bonferroni. (**e**) A non-linear regression analysis of serial samples (patients that had two or more samples during the study) is shown. Non-linear regression graph shows individual patient trajectories with dotted lines and 90% CI as filled space. ROC curve-analysis for ICU mortality prediction according to (**f**) highest or (**g**) earliest SARS-CoV-2 copy number is shown. AUC = area under the ROC curve; ns = non-significant; * = *p* < 0.05; ** = *p* < 0.01; **** = *p* < 0.0001.

We quantified SARS-CoV-2 copy numbers in serum samples and compared groups of increasing severity of clinical symptoms and disease outcomes. Hence, we compared non-hospitalized patients, symptomatic hospitalized patients, and severely ill patients on mechanical ventilation, subdividing the last group into survivor and non-survivor subgroups. No significant differences were found between the samples from the non-hospitalized patients (outpatient SARS-CoV-2 positive patients detected by nasopharyngeal swab without self-reported clinical manifestations) and the symptomatic patients (hospitalized severe COVID-19 patients requiring supplementary O_2_, but without fulfilling criteria for mechanical ventilation or ICU admission). Serum SARS-CoV-2 copy numbers were significantly higher in the ICU samples than in the symptomatic subjects. When classified by ICU mortality outcome (survival at discharge vs. death during ICU stay), serum samples from the deceased patients had higher SARS-CoV-2 copy numbers than among the survivors (Figure 1a). The same difference was present when comparing the highest viral copy number per patient and the earliest viral copy number available per patient between survivors and non-survivors (Figure 1b,c). To follow up on the SARS-CoV-2 viral copy numbers during the ICU stay, we categorized the samples according to their time of sampling post-intubation (PI). Immediate (first 72 h PI), intermediate (between 72 and 120 h PI) and late (sampling performed more than 120 h PI) intervals were defined and compared. A higher viral copy number was found in the non-survivor group at the immediate interval (Figure 1d), but not at the later time points. Furthermore, we performed a non-linear regression model on serial samples (two or more) obtained from individual ICU patients, but observed no substantial differences in their kinetics (Figure 1e). Finally, we executed a receiver operating characteristic (ROC) curve analysis on the highest and earliest SARS-CoV-2 copy number per patient for its survival classification performance (Figure 1f,g). Both evaluations were statistically significant (*p* = 0.0069 for the highest, *p* = 0.0142 for the earliest). 

**Figure 2:**
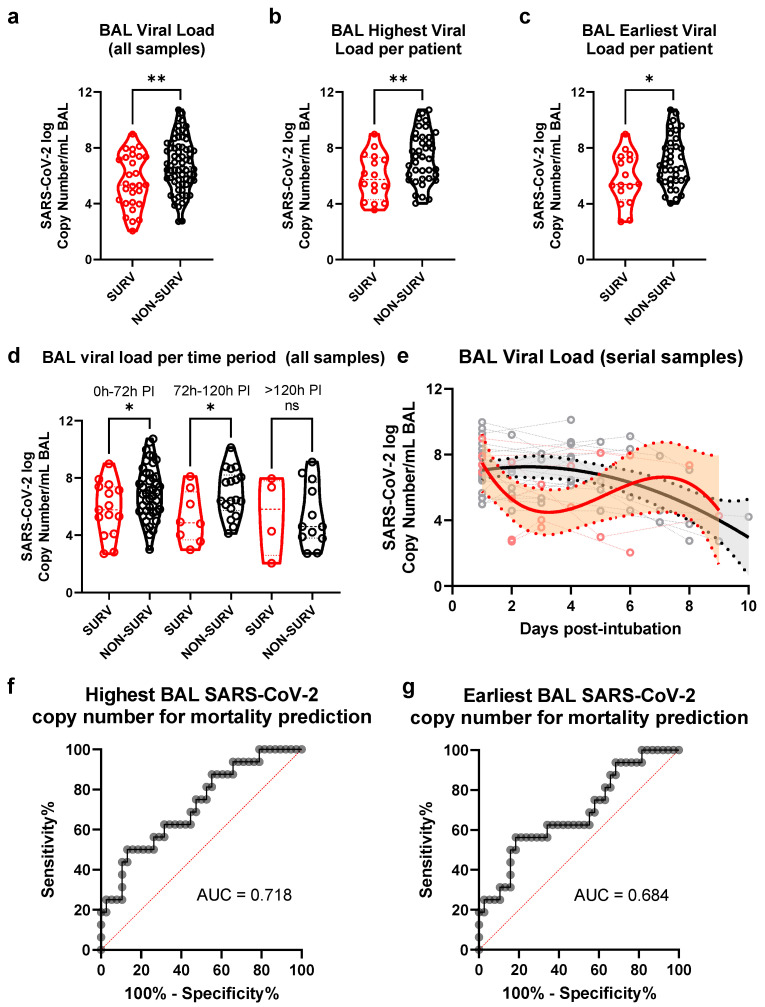
SARS-CoV-2 viral load in BAL samples from SARS-CoV-2 ICU patients. Bronchoalveolar lavage was performed in patients admitted to the ICU with COVID-19 associated acute respiratory failure diagnosis. In some patients, BAL sampling was performed at multiple time points. A pooled analysis including all samples was performed according to ICU survival in (**a**). Analysis of samples with the highest viral load per patient (**b**) and earliest sample available per patient (**c**) is shown. A pooled analysis divided by sampling period after intubation (PI) was performed (**d**). A non-linear regression analysis of serial samples (patients that had two or more samples during the study) is shown (**e**). A violin plot graph showing median and quartiles of SARS-CoV-2 copy numbers, as well as all data points (circle) was used. Non-linear regression graph shows individual patient trajectories with dotted lines and 90% CI as filled space. ROC curve-analysis for ICU mortality prediction according to (**f**) highest or (**g**) earliest SARS-CoV-2 copy number is shown. AUC = area under the ROC curve; ns = non-significant; * = *p* < 0.05; ** = *p* < 0.01.

RNA was extracted from the BAL samples and analyzed by RT-qPCR to define SARS-CoV-2 copy numbers. Samples from non-survivor ICU patients had higher SARS-CoV-2 copy numbers than the survivor group (Figure 2a). The same difference was present when the highest viral copy number per patient and the earliest viral copy number available per patient between survivors and non-survivors were compared (Figure 2b,c). When classified by the time of sampling, a higher viral copy number was found among the non-survivors in the immediate and intermediate PI interval (Figure 2d). A non-linear regression model was performed on serial samples (two or more) obtained from individual ICU patients corroborating a slightly higher early virus titer among the deceased (Figure 1e). Finally, we executed a ROC curve analysis on the highest and earliest SARS-CoV-2 copy number per patient for its survival classification performance (Figure 1f,g) and observed a statistically significant distribution in both cases (*p* = 0.012 for highest, *p* = 0.034 for earliest).

In order to analyze the differences in viral load from the pulmonary and systemic compartments between the second (potential pre-alpha variants) and third wave (potential alpha variant), we classified ICU patients according to their time of admission (Table 3). It is important to mention that the treatment protocols for ICU patients did not change between the two waves. Statistically significant differences in BAL and serum SARS-CoV-2 copy numbers were found between the second and third waves. 

### 3.3. Detection of SARS-CoV-2 Variants and Comparison of Viral Load

**Figure 3:**
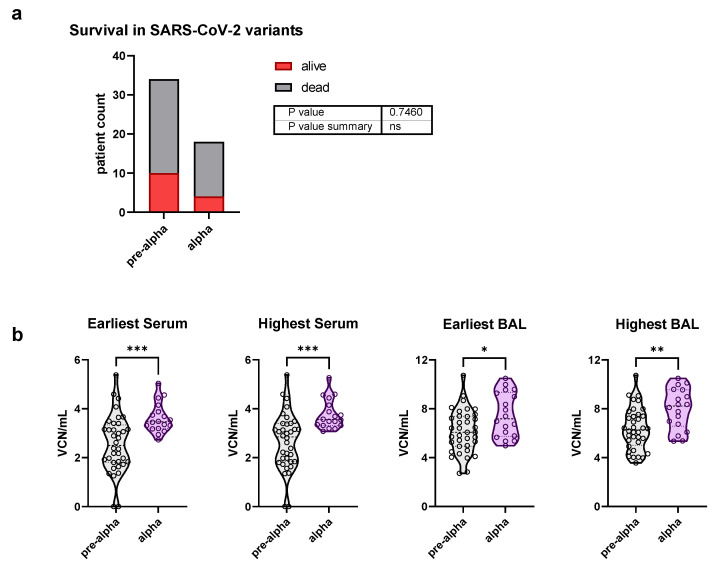
SARS-CoV-2 viral load of pre-alpha and alpha infected patients. SARS-CoV-2 variants in patient samples were identified by RT-qPCR. A Fisher’s exact test was performed to compare the outcome based on infections by SARS-CoV-2 pre-alpha and alpha VoC (**a**). A pooled analysis including all samples was performed according to SARS-CoV-2 pre alpha and alpha infection. Analysis of samples with the earliest and highest viral load per patient in serum and BAL samples is shown (**b**). Violin plot graphs showing medians (dashed lines) and quartiles (dotted lines) of SARS-CoV-2 copy numbers, as well as individual data points (circles) was used. Two patients from the second wave were excluded based on inconclusive variant detection. * = *p* < 0.05; ** = *p* < 0.01; *** = *p* < 0.001.

We considered that differences in viral loads could have been a result of differences in viral genotypes, particularly as the patients in the third wave could have been infected with the variant of concern (VoC) α or a pre-α viral variant. We identified the SARS-CoV-2 variants by RT-PCR, specifically for mutations in the spike protein. In the second wave, 93.94% of patients (10 survivors; 21 non-survivors) were infected by pre-α SARS-CoV-2, while in two surviving patients (6.06%), the results were inconclusive. In the third wave, 14.29% of patients (three survivors) were classified as pre-α SARS-CoV-2, and 85.71% of patients (4 survivors; 14 non-survivors) were alpha-infected patients. A Fisher’s exact test showed no significant association between variant infection and mortality (Figure 3a), showing that disease outcome was not severely affected by the underlying variant. A comparison of viral load from pre-alpha and alpha infected patients, however, showed a significantly higher viral load in ICU patients who were infected by SARS-CoV-2 VoC α (Figure 3b).

### 3.4. Correlations of SARS-CoV-2 Viral Loads with Age and Severity Score at Admission

**Figure 4:**
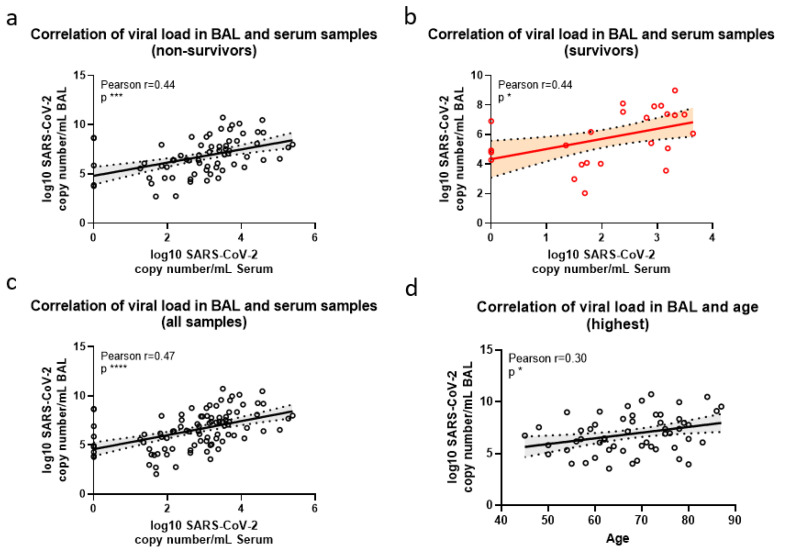
Correlation of SARS-CoV-2 viral load in BAL and serum samples of severe COVID-19 ICU patients. BAL and serum samples were obtained from ICU patients with severe COVID-19 at the same time points. A linear regression and Pearson correlation analysis was performed on the viral load in BAL and serum samples at each time point for deceased (**a**), survivors (**b**) and of all samples (**c**) as well as the viral load in BAL and age with highest samples per patient (**d**). Dotted lines show the 95% confidence interval. * = *p* < 0.05; *** = *p* < 0.001; **** = *p* < 0.0001.

We correlated SARS-CoV-2 RNA copy numbers in the BAL samples to viral RNA loads in serum samples collected at similar time points. A significant correlation was observed in the group of non-survivors (*p* = 0.0002, Figure 4a), survivors (*p* = 0.0315, Figure 4b) and when both non-survivors and survivors were pooled (*p* =< 0.0001, Figure 4c). We also correlated BAL or serum viral loads to age, either by focusing on the earliest sample or by the highest-load sample. Only a correlation between age and the highest BAL viral load could be observed (*p* = 0.0251, Figure 4d), whereas no correlation between age and serum samples was ascertained (not shown). 

**Figure 5:**
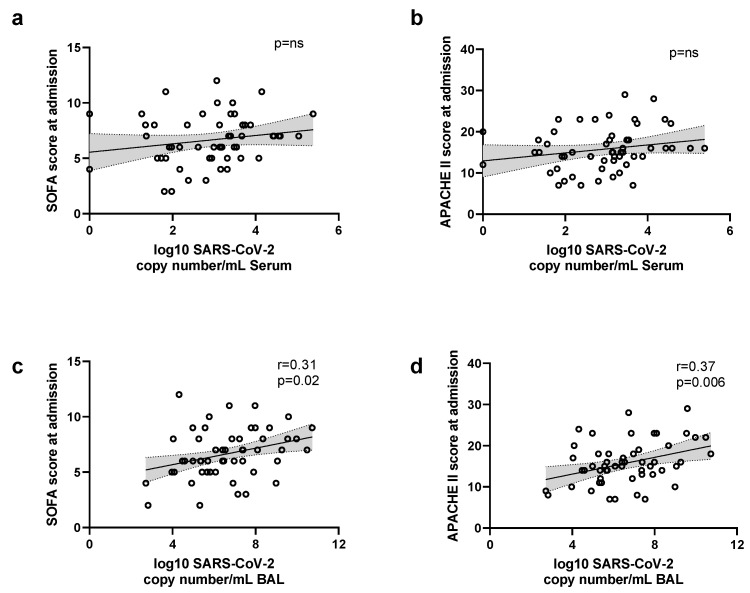
Correlation of earliest SARS-CoV-2 viral load in BAL and serum samples with severity scores at ICU admission. A linear regression and Pearson correlation analysis was performed between the earliest viral load in serum samples with SOFA (**a**) and APACHE II (**b**). Same analysis was performed between the earliest viral load in BAL samples with SOFA (**c**) and APACHE II (**d**). Dotted lines show the 95% CI.

To investigate the association of SARS-CoV-2 copy numbers with ICU severity scoring at admission, a correlation was performed using the earliest viral load and APACHE II or SOFA scores (Figure 5). No significant correlation was found between ICU scores and serum samples, while a weak correlation was present when BAL samples were analyzed.

To confirm the significance of the association of SARS-CoV-2 copy numbers with mortality, we performed a multivariate analysis of the earliest and highest SARS-CoV-2 copy numbers in the serum and BAL samples, including clinical and laboratory variables at admission identified in Table 1. We determined the best cut-off values for mortality classification according to Youden’s J analysis (Supplementary File S2). Due to linear dependence among PaO_2_/FiO_2_, SOFA and APACHE-II (PaO_2_ and FiO_2_ are included in SOFA and APACHE-II scores), we decided to use PaO_2_/FiO_2_ due to its superior performance in univariate ROC analysis (AUC = 0.93). Using this model, all viral copy number quantifications, except the earliest BAL, were identified as significant predictors of mortality (Table 4). 

## 4. Discussion

In order to establish an association to illness severity, copy numbers have been measured in various respiratory tract samples such as sputum and tracheal aspirates, indicating an association between elevated virus titers and mortality in some, but not all studies [17,18,19]. Lung pathology dominates COVID-19 pathogenesis and SARS-CoV-2 replication in the lungs can be assessed directly by measuring viral loads in the BAL fluid [14]. Hence, we analyzed BAL samples and identified an association between the BAL viral copy numbers and clinical outcomes (severity and mortality) in critically ill ARDS COVID-19 patients. Remarkably, we found that early BAL viral load correlates to ICU score tools of acute multiorgan dysfunction (SOFA) and mortality prognosis (APACHE II). This finding suggests that COVID-19 organ damage is associated with virus replication/permanence within the alveolar compartment at a relatively early time during severe pulmonary deterioration. While BAL samples have been considered for diagnostic purposes in early COVID studies [20,21], the association between BAL copy numbers and/or prognosis was not addressed at the time. To the best of our knowledge, only one brief report investigated the correlation between BAL viral copy number and disease severity in 14 ICU patients [22]. Hence, our study is the first analysis of COVID-19 RNA quantity in the lungs of a larger, better characterized, ICU cohort. 

We also noticed a significant, but not tight, correlation between BAL and serum viral loads in paired analysis. This might argue that serum RNA quantification could be used as a proxy for the more challenging alveolar specimen, as BAL sampling requires specialized training and operator-dependent variability may occur. Moreover, the physiological changes associated with sample timing and concomitant pulmonary conditions (increased mucus in chronic respiratory disorders, simultaneous ventilator-associated pneumonia) may contribute to the variability in RNA stability upon sampling. BAL samples are highly contagious specimens, which increases the risk for the medical and laboratory personnel. Therefore, correlates of lung virus load in serum, which are more readily accessible, may be an alternative and somewhat neglected prognostic marker of COVID-19 disease severity. 

We detected SARS-CoV-2 RNA in 86% of our serum ICU samples. A wide range of SARS-CoV-2 RNAemia has been reported in COVID-19 [23,24,25] (ranging from 2.8% [26] to 78.6% in hospitalized patients [27]), and viral RNA presence in serum had been associated with the more severe phenotype of the disease [23,25,27,28,29,30,31,32,33,34,35]. Our results confirm this finding and further expand on the relationship between viral load quantification and mortality, as higher peak SARS-CoV-2 serum copy numbers were found in the non-survival group. While most studies focus on samples acquired at the onset of symptoms or initial hospital admission (resulting in contradictory results on its association to mortality [36,37,38]), we report that early serum viral load after intubation may have a role as a predictor for ICU mortality. As initiation of invasive ventilatory support is a robust indicator of clinical deterioration, this may represent an improved time-point for standardization. Given the widespread availability of SARS-CoV-2 quantification in the clinical setting and relatively rapid results, serum viral copy numbers may function as a stratification method to distinguish those critically ill patients that would benefit from early advanced therapy.

We studied a critically ill COVID-19 patient cohort undergoing invasive mechanical ventilation (IMV) support, which presented a mortality rate of 70.9%. Although a wide range of ICU survival rates have been reported [39,40,41], and mortality is heavily dependent on a patient’s age [8], sex, and comorbidities [42], fatal outcomes were particularly high in our study population in comparison to other European ICU reports on patients requiring IMV (36%, 95% CI 24–48%) [43]. The high mortality was likely a result of several adverse conditions. On one hand, clinical capacity was severely strained during the pandemic peaks and the Croatian healthcare system has a low ICU accessibility index, which is associated with worse COVID-19 outcomes [44]. On the other hand, the selection bias in a regional third-level specialized referral center likely drove the assortment of a subgroup of rapidly progressing and/or severely affected COVID-19 patients. Supporting this explanation, the SOFA and APACHE-II scores at admission (which are validated ICU mortality estimation tools) were markedly high in our cohort. Using a proposed APACHE-II cut-off score for mortality risk in severe COVID-19 [45], predicted mortality was even higher than observed (79.62% vs. 70.9%), arguing for a particularly ill population at admission. Regardless of the underlying cause, our study represents a sample of COVID-19 ICU patients during pandemic peaks, and thus bears relevance for real-life challenging clinical situations.

Well-established ICU scoring systems that evaluate organ-damage and preexisting conditions have been proved useful in predicting mortality in COVID-19 critically ill patients. To our surprise, after evaluation of the earliest sample available, only SARS-CoV-2 copy numbers in the BAL correlated with SOFA or APACHE II, arguing in favor of an independent role for serum SARS-CoV-2 copy numbers in identifying fatal outcomes. It is important to note that for an adequate assessment of the clinical relevance of SARS-CoV-2 copy numbers as a predictor for mortality, the presence of other variables such as age, comorbidities, and even variant type should be considered for a more realistic evaluation in larger validation cohorts. Using a multivariable analysis of mortality outcome that included age, PaO_2_/FiO_2_ and CRP, an independent significance was found only for peak, but not for the earliest SARS-CoV-2 RNA quantification in the BALs. On the other hand, serum viral copy numbers, both the earliest and highest values, maintained their significance after correcting for these possible confounding factors.

Our study possesses several limitations. Sampling occurred in late 2020 and early 2021, prior to the onset of the new delta variant. Additional studies on contemporary samples are required to test whether the delta virus behavior will match the one described in this work. Interestingly, a non-significant trend towards higher viral load was observed in the third wave, which coincided with the emergence of the alpha variant. Therefore, we tested the genetic background of the viruses and compared virus titers on the VoC α with the pre-α SARS-CoV-2 and noticed a significantly higher virus titer in patients infected with the VoC α. To our knowledge, this is the first direct evidence that a variant was associated with elevated virus yields in the lung. However, due to the low count of survivors in the VoC α infected subcohort, we did not assess if the titers differed among subgroups classified according to virus genetic backgrounds. Another limitation is that the sampling was performed during two waves of the virus pandemic, when the hospital system was under severe strain. Of note, no changes in management guidelines took places between the waves. It remains unclear whether patients admitted to the hospital at times of lesser incidence would behave similarly, as the availability of personnel and hospital care may influence patient survival. Despite these limitations, our results provide a direct insight into virus quantity in the pulmonary and systemic compartments, which critically determines pathogenicity and the clinical outcome of COVID-19 disease.

## 5. Conclusions

To conclude, we investigated the SARS-CoV-2 viral copy numbers of the systemic and pulmonary compartments in COVID-19 critically ill patients by analyzing serum and BAL fluid during their ICU stay. We found a significant correlation between both compartments at similar time points. BAL viral copy numbers were significantly correlated to disease severity score at ICU admission, but fatal outcome was associated with higher copy numbers at ICU admission in both the serum and BAL. Our study shows that BAL SARS-CoV-2 viral load measurement in critically ill patients is feasible and may provide additional information for clinical evaluation and patient stratification.

## Figures and Tables

**Table 1:** Demographic and clinical characteristics of the patients included in this study, classified by their ICU stay and outcome.

	ICU Non-Survival (*n* = 38)	ICU Survival (*n* = 16)	Non-ICU Symptomatic (*n* = 16)	*p* between Non-Survival and Survival Groups)
**Demographic characteristics**				
Female %	31.58	25	25	ns ^a^
Age in years (Mean ± SD)	71 ± 9.87	64 ± 10.15	70 ± 14.25	0.023 ^b^
Sample per patient	1.87 ± 0.93	1.94 ± 0.93	1	ns ^c^
**Clinical characteristics**				
PaO_2_/FiO_2_ at ICU admission (Median, p25–p75)	93 (83.5–109.8)	159.5 (128–191.8)	-	<0.0001 ^c^
Moderate-Severe ARDS at admission (%)	100	75	-	0.0058 ^a^
SOFA score at admission (Median, p25–p75)	7 (6–9)	4.5 (3.25–5)	-	<0.0001 ^c^
APACHE II score at admission (Median, p25–p75)	16 (15–22)	10.5 (8.25–13)	-	<0.0001 ^c^
Fever at admission (%)	42.11	31.25	43.75	ns ^a^
ICU stay length in days (Median, p25–p75)	11 (6–14)	15.5 (13.25–17.75)	-	0.0016 ^c^
SARS-CoV-2 Immunization started (%)	0	0	6.25	ns ^a^
**Comorbidities**				
Coronary heart disease (%)	23.68	12.5	18.75	ns ^a^
Hypertension (%)	76.32	68.75	62.5	ns ^a^
Diabetes (%)	39.47	18.75	25	ns ^a^
Obesity (%)	26.32	43.75	12.5	ns ^a^
Cancer (%)	5.26	12.5	12.5	ns ^a^
Chronic respiratory disease (%)	15.79	18.75	6.25	ns ^a^
Immunosuppression (%)	2.63	0	0	ns ^a^
Number of comorbidities (Median, p25–p75)	2 (1–3)	2 (1–2.75)	1 (0–2.75)	ns ^c^
**Laboratory Markers** **at Admission**				
WBC c/uL × 10^6^ (Median, p25–p75)	11.10 (8.8–13.3)	12.35 (10.93–15.23)	7.4 (6.25–12.85)	ns ^c^
Hemoglobin g/L (mean ± SD)	123.7 ± 20.28	129.9 ± 15.2	126.81 ± 26.75	ns ^b^
CRP mg/dL (mean ± SD)	157.9 ± 77.98	93.66 ± 74.38	96.6 ± 78.48	0.0078 ^b^
**Treatment during ICU** **hospital stay**				
Days on mechanical ventilation (Median, p25–p75)	11 (5.75–14)	8 (5.25–12)	-	ns ^c^
Days of Supplementary O_2_ administration (Mean ± SD)	-	-	7.13 ± 7.44	-
Shock (%)	65.79	6.25	6.25	<0.0001 ^a^
Days on vasopressors and/or inotropics (Median, p25–p75)	4 (2–5.25)	1 (0–3.75)	0	0.0093 ^c^
Renal Replacement therapy use (%)	5.26	12.5	0	ns ^a^
Ventilator-associated pneumonia (%)	63.16	43.75	-	ns ^a^
Catheter-associated bloodstream infection (%)	15.79	0	0	ns ^a^

ns = non-significant. ^a^ calculated by Fisher’s exact test. ^b^ calculated by unpaired t test with Welch’s correction. ^c^ calculated by Mann–Whitney test.

**Table 2:** Demographic and clinical characteristics of patients included in this study according to their time of admission (second vs. third Wave).

	Second Wave *n* = 33	Third Wave *n* = 21	*p*
Age in years (Mean ± SD)	66.94 ± 9.2	71.67 ± 11.71	ns ^1^
Female %	30.30	28.57	ns
Number of Comorbidities per patient (median, p25–p75)	2 (1.5–3)	1 (1–3)	ns
PaO_2_/FiO_2_ at ICU admission (Median, p25–p75)	97 (85.5–158)	119 (89–132)	ns
SOFA score at admission	6 (5–8)	7 (6–9)	0.028
CRP mg/dL at admission (Mean ± SD)	135.5 ± 75.21	144.1 ± 92.85	ns
Days on mechanical ventilation (median, p25–p75)	10 (5.5–13.5)	10 (5–15)	ns
Survival at ICU discharge (%)	36.36	19.05	ns

^1^ ns = non-significant.

**Table 3:** Second vs. third wave viral load (log converted) (mean ± SD).

	Second Wave	Third Wave	*p*
Highest Serum Viral Load	2.67 (±1.05)	3.59 (±0.99)	0.0027
Earliest Serum Viral Load	2.57 (±1.07)	3.46 (±0.97)	0.0035
Highest BAL Viral Load	6.27 (±1.62)	7.89 (±1.72)	0.0016
Earliest BAL Viral Load	6.09 (1.69)	7.43 (±1.79)	0.0102

**Table 4:** Multivariable analysis of SARS-CoV-2 copy numbers with relation to ICU survival.

Variable	Odds Ratio	95%CI	*p*
Age > 76 years	1.50	0.11 to 40.77	0.77
CRP > 153.2 mg/dL	6.75	0.79 to 87.84	0.10
PaO_2_/FiO_2_ < 118	30.17	4.85 to 325.7	0.001
Earliest Serum Viral Load > 3.32 log_10_ copy number/mL	19.30	1.86 to 533.1	0.03
Age > 76 years	1.97	0.16 to 50.88	0.62
CRP > 153.2 mg/dL	6.57	0.82 to 81.87	0.10
PaO_2_/FiO_2_ < 118	29.41	4.83 to 318.2	0.001
Highest Serum Viral Load > 3.32 log_10_ copy number/mL	9.76	1.27 to 122.0	0.04
Age > 76 years	2.93	0.24 to 74.30	0.43
CRP > 153.2 mg/dL	11.34	1.32 to 186.6	0.046
PaO_2_/FiO_2_ < 118	20.46	3.62 to 196.8	0.002
Earliest BAL Viral Load > 5.42 log_10_ copy number/mL	6.00	0.86 to 66.75	0.09
Age > 76 years	5.52	0.40 to 171.4	0.24
CRP > 153.2 mg/dL	10.84	1.43 to 129.6	0.03
PaO_2_/FiO_2_ < 118	23.32	3.84 to 253.5	0.002
Highest BAL Viral Load > 7.54 log_10_ copy number/mL	11.22	1.34 to 158.8	0.04

## Data Availability

The data presented in this study are available on request from the corresponding author.

## Supplementary Materials

The following supporting information can be downloaded at: https://www.mdpi.com/article/10.3390/v14061292/s1, Supplementary File S1: COVID-19 ICU Management Guidelines from the Rijeka Medical Center; Supplementary File S2: Youden’s J statistics of mortality-dependent ROC curves.
