# Supplementary material for: SARS-CoV-2 Viral Load in the Pulmonary Compartment of Critically Ill COVID-19 Patients Correlates with Viral Serum Load and Fatal Outcomes
Source: Viruses. 2022 Jun 14;14(6):1292. doi: 10.3390/v14061292 (PMC9228931; doi:10.3390/v14061292)
Supplement: Supplementary file 1 [file viruses-14-01292-s001.zip › Supplementary Files/Supplementary File 1.pdf]

Supplementary File 1

COVID-19 ICU Management Guidelines from the Rijeka Medical Center

- Indication for intubation and invasive mechanical ventilation: a. Rapid clinical deterioration in the last two hours (increased hypercapnia, respiratory effort, deterioration of mental state) and b. failure or intolerance of non-invasive ventilation or failure to improve after 30 minutes on high flow nasal cannulae.

- Initial mechanical ventilatory support settings: a. Initial lung recruitment maneuver of 15 s at 40 mmHg, b. Volume controlled ventilation with tidal volume (V_t) at 4 mL/ kg of IBW (ideal body weight); c. Respiratory rate 20 per minute. c. Plateau pressure: 22 cm H_2O; Positive end-expiratory pressure (PEEP) at 10 mmHg. d. Initial Fraction of inspired oxygen (FiO_2) at 0.5.

- In case of requiring higher ventilatory support, a step-by-step increase was done: I. Increase PEEP to 15 mmHg; II. Increase V_t to 6 mL/kg; III. Increase FiO_2 to 1; IV: Increase respiratory frequency up to 30 per minute

- If there is no significant improvement after 6 hours, prone ventilation is indicated for minimum of 12 hours per day (usually 18-20 hours). When improving, first lower FiO2 to 0.35, then PEEP and frequency, and initiate weaning. After extubation, all patients start with physical and respiratory therapy with early mobilization.

- Hemodynamic support: Noradrenaline is used to achieve a mean arterial pressure ≥ 60 mmHg. A conservative fluid approach was followed to keep a zero balance (intake equal to amount). Maintain diuresis ≥ 50 mL/. Diuretic support as necessary.

- Analgesia and sedation: Supine ventilation position: propofol + sufentanil or dexmedetomidine + sufentanil. Prone ventilation position: propofol + sufentanil + rocuronium. Midazolam is avoided.
-
- Systemic Therapy: If the patient did not previously receive systemic steroid treatment: methylprednisolone 1 mg/kg for the first 3 days and 0.5 mg/kg for the next 3 days. If steroid treatment with dexamethasone was started, continue with dexamethasone 6-8 mg until the seventh day from the start of therapy. Thromboprophylaxis was achieved by enoxaparin (2 x 1 mg/kg of body weight). No empirical antibiotic therapy at admission, therapy is directed by blood, urinary and respiratory cultures in concordance with laboratory findings and clinical condition. Remdesivir is not indicated.

- Nutrition: Early enteral nutrition is started at 30 mL/h via nasogastric tube, increased at the third day of ICU stay to 50-60 mL/h. While prone ventilation is conducted, enteral nutrition is decreased to 10 mL/h. Tolerate glycaemia up to 12 mmol/L without the addition of insulin. Pantoprazole should only be given to invasive mechanically ventilated patients.
