# Supplementary material for: SARS-CoV-2 Viral Load in the Pulmonary Compartment of Critically Ill COVID-19 Patients Correlates with Viral Serum Load and Fatal Outcomes
Source: Viruses. 2022 Jun 14;14(6):1292. doi: 10.3390/v14061292 (PMC9228931; doi:10.3390/v14061292)

Supplementary File 2

Youden's J statistics of mortality-dependent ROC curves

Clinical variables included are those that differed between Survivors and Non Survivors at admission with statistical significance

AGE

Youden index J	0.3059
Associated criterion	76
Sensitivity	36.84
Specificity	93.75

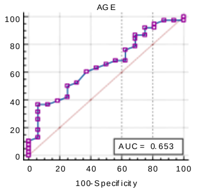

APACHE II

Youden index J	0.7599
Associated criterion	13
Sensitivity	94.74
Specificity	81.25

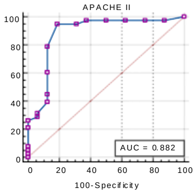

SOFA

Youden index J	0.7336
Associated criterion	5
Sensitivity	92.11
Specificity	81.25

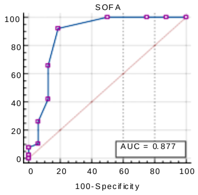

PaO_2/FiO_2

Youden index J	0.6908
Associated criterion	118
Sensitivity	81.58
Specificity	87.5

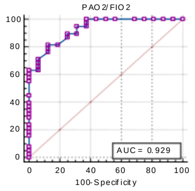

C Reactive Protein

Youden index J	0.5066
Associated criterion	153.2
Sensitivity	63.16
Specificity	87.5

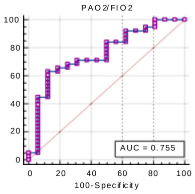

EARLIEST BAL VIRAL COPY NUMBER

Youden index J	0.2895
Associated criterion	5.42
Sensitivity	78.95
Specificity	50

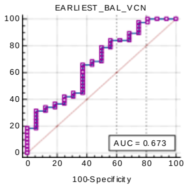

HIGHEST BAL VIRAL COPY NUMBER

Youden index J	0.3224
Associated criterion	7.54
Sensitivity	44.74
Specificity	87.5

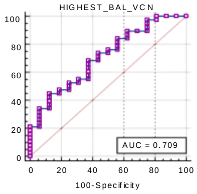

EARLIEST SERUM VIRAL COPY NUMBER

Youden index J	0.4375
Associated criterion	3.32
Sensitivity	50
Specificity	93.75

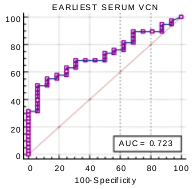

HIGHEST SERUM VIRAL COPY NUMBER

Youden index J	0.4276
Associated criterion	3.32
Sensitivity	55.26
Specificity	87.5

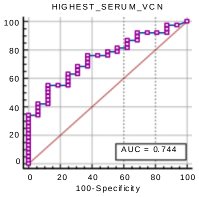

Supplement: Supplementary file 1 [file viruses-14-01292-s001.zip › Supplementary Files/Supplementary File 2.pdf]
